# An effective cytokine adjuvant vaccine induces autologous T-cell response against colon cancer in an animal model

**DOI:** 10.1186/s12865-016-0172-x

**Published:** 2016-09-26

**Authors:** Huanyu Ju, Wenjing Xing, Jinfeng Yang, Yang Zheng, Xiuzhi Jia, Benning Zhang, Huan Ren

**Affiliations:** 1 Department of Immunology, Harbin Medical University, 157 Baojian Road, Harbin, 150081 China; 2 Infection and Immunity, Key Laboratory of Heilongjiang Province, Harbin, 150081 China

**Keywords:** Colon cancer, CT26.WT, Cytokine adjuvant, Immunotherapy, Tumor vaccine

## Abstract

**Background:**

Despite recent advances in early detection and improvements in chemotherapy for colon cancer, the patients still face poor prognosis of postoperative recurrence and metastasis, the median survival for patients with metastatic colorectal cancer is approximately 22–24 months. Some immunotherapeutic approaches had been attempted in colon cancer patients to significantly increase overall survival. A vaccine based approach has shown a novel direction for colon cancer prevention and therapy.

**Methods:**

In this study, the experiments were designed including prevention and therapeutic stages in order to attain effect against tumor recurrence in clinical settings. The anti-tumor efficacy of a novel cytokine adjuvant vaccine that contained cytokines GM-CSF and IL-2 and inactivated colon CT26.WT whole cell antigen was evaluated in BALB/c mouse tumor models by measuring tumor growth post vaccination and the survival time of tumor-bearing mice, analyzing the expression and distribution of CD4, CD8, CD11c, CD80, CD86 and CD83 positive cells in control and treated mice by flow cytometry and immunochemistry. The tumor-specific cytotoxic T cells (CTL) were analyzed by tumor proliferation and the lactic dehydrogenates (LDH) release assays. IFN-γ, IL-2 and GM-CSF secretion in serum was assayed by ELISA.

**Results:**

Our results suggested that cytokine adjuvant vaccine significantly inhibited tumor growth and extended the survival period at least 160d. It was found that the levels of CD8 + T and the tumor-specific cytotoxicity were significantly higher in prevention and treatment group vaccinated by cytokine adjuvant vaccine. CD8 + T cells play a key role in anti-tumor response.

**Conclusions:**

The novel GM-CSF and IL-2 based adjuvant vaccine effectively activated autologous T-cell response and represented a promising immunotherapeutic approach for patients with colon cancer.

**Electronic supplementary material:**

The online version of this article (doi:10.1186/s12865-016-0172-x) contains supplementary material, which is available to authorized users.

## Background

Colon cancer is a common malignant tumor of the digestive tract [1]. It remains a challenge to treat advanced colon cancer due to postoperative recurrence and metastasis. Currently, surgical resection of primary colorectal lesions combined with adjuvant chemotherapy and radiation continues as the mainstay of therapy [2]. Unfortunately, around 30 % of colorectal carcinoma patients are diagnosed with metastatic disease at initial presentation, and an additional 25–30 % subsequently develops metastatic disease [3, 4]. Despite recent advances in early detection and improvements in chemotherapy, the median survival for patients with metastatic colorectal cancer is approximately 22–24 months, with 5-year survival less than 5 %. Recently, additional immunotherapeutic approaches such as cytokine, cytokine-induced killers (CIK), mAb and tumor vaccine therapy in patients with advanced colon cancer significantly increase overall survival [5–8]. Moreover, these approaches also showed to be safe and effective. Amongst these, therapeutic tumor vaccines showed some promise. For example, preclinical studies demonstrated an enhanced antitumor activity of granulocyte–macrophage colony-stimulating factor (GM-CSF) or IL-2 producing murine colon tumor cell vaccines [9, 10] and each cytokine have been used as an adjuvant with cancer vaccines [11, 12]. Nevertheless, so far the treatment with vaccines failed to significantly improve the 5-year survival of patients with colon cancer. Therefore, a new adjuvant therapeutic vaccine is needed.

GM-CSF is an important growth and differentiation factor for dendritic cells, which are potent antigen-presenting cells that can take up cellular proteins as tumor antigens [13]. For an effective antitumor response, preclinical studies suggested that GM-CSF secretion occur at the site of vaccination and high levels of the cytokine must be sustained for several days [14, 15]. GM-CSF-producing vaccines, together with autologous or allogeneic tumor cells, were frequently tested in preclinical and clinical studies [16–18], and antitumor responses were observed in various models such as clinical trials with pancreatic cancer patients [19–21]. Thus, a GM-CSF producing bystander cell line significantly improved the feasibility and efficacy of autologous or allogeneic tumor vaccines in human studies. IL-2 is a principle cytokine responsible for the differentiation of effector T cells. Relevant studies indicated that targeting IL-2 to tumor cells facilitated their elimination via enhanced T cell activation [9]. Therefore, IL-2 has the potential to aid in eliciting anti-tumor responses. Several studies suggested that IL-2 can be used in tumor vaccines, where it enhanced the antitumor response against several cancers [22, 23].

In the present study, we evaluated the efficacy of cytokine adjuvant (recombinant mouse GM-CSF and IL-2) vaccine pulsed with inactivated CT26.WT colon tumor cells via measuring the tumor-specific CTL activity and associated anti-tumor effect in a colon cancer model.

## Methods

### Animals and cells

Female and male BALB/c mice, 6 weeks old were purchased from the Beijing Experiment Animal Center, Chinese Academy of Sciences (Beijing, China). 130 mice were used for preliminary experiment, and 240 mice were used for formal experiment. All mice were kept under pathogen-free conditions in the animal center of the Harbin Medical University (Harbin, China). CT26.WT, a mouse colon adenocarcinoma cell line was purchased from the Shanghai Cell Biology Institutes, Chinese Academy of Sciences (Shanghai, China) and was cultured in RPMI medium 1640 (Hyclone, China) containing 10 % heat-inactivated fetal calf serum (FCS), 2 mmol/L glutamine, penicillin G (100U/mL), and streptomycin (100 μg/mL) at 37 °C in a humidified incubator supplemented with 5 % CO_2_.

### Tumor model and vaccine production and immunization

To establish the tumor model, CT26.WT tumor cells (5 × 10^5^cells per mouse) were injected subcutaneously into the neck of BALB/c mice. Tumors were observed after 8 days of cells inoculation in control mice. The cytokine adjuvant vaccine consisted of the following components: (1) GM-CSF & IL-2 cytokines (R & D, USA) adjuvants prepared by heparin and HSA (human serum albumin) package (4500 IU/30 μl) and (2) CT26.WT tumor cells that were inactivated by mitomycin C (MMC) (Fuji Plant, Japan) for 2 h and then mechanically digested and counted. Finally, each vaccine dose was constituted by mixing 30 μl of each, GM-CSF and IL-2 as well as 40 μl of inactivated CT26.WT tumor cells (1 × 10^6^ cells). The cytokine adjuvant vaccine was immunization subcutaneously into the underarm and foot-pad of BALB/c mice for different times.

### Lymphocytes isolation and FACS analysis

Lymphocyte cells were isolated from lymph nodes by manual dissociation and were suspended in Hanks buffer (Sigma, China). T cells were further purified by CD3 microBeads (Miltenyi Biotech, Germany) and counted. Splenocytes were isolated and treated with ACK red blood cell lysis buffer (Invitrogen, China) following manual dissociation, and then the similar protocol was used for isolation of as described above. Finally, lymphocytes (1 × 10^6^) were stained with FITC-conjugated mAbs against mouse CD4 or CD8 and PerCP-Cy™5.5-conjugated against mouse CD3, all purchased from BD Pharmingen (BD, USA). Next, these stained cells were analyzed using a FACS Caliber flow cytometer (BD, USA) and the Cell Quest software package (BD, USA).

### Cytotoxic T lymphocyte activity assay and cytokines detection

To confirm the tumor-specific activity of cytotoxic T lymphocytes (CTLs), T lymphocytes were extracted at different time points. CD3^+^ T cells were isolated using CD3 microBeads (Miltenyi Biotech, Germany). CD8^+^ T cells were obtained by flow cytometry using anti-CD4 antibody by negative selection. Purified CD8^+^T cells from each group of mice were cultured in RPMI 1640 medium containing 10 % FCS. Next, effector T cells were mixed and incubated with target CT26.WT cells in a 96-well plates (*E: T*, 50:1) optimization for 24 h. Thereafter, the supernatant from each well was collected and the cytolytic activity was measured by a LDH cytotoxicity detection kit (Nanjing jiancheng, China). For measuring CTL activity, we used the MTT cell viability detection kit (Sigma, China). The target CT26.WT cells were added into 96 well plates over night, and effector T cells suspensions were added in *E: T* ratio of 50:1 optimization. The serum samples of each group of mice were collected and stored at −80 °C. The cytokines, IFN-γ, IL-2 and GM-CSF levels were detected by using mouse cytokine ELISA Kit (RayBio®, USA).

### Immunohistochemical staining analysis

Lymph nodes and tumors from each group were collected, fixed, embedded in paraffin, and sections stained for CD4, CD8, CD11c, CD80, CD86 and CD83 markers (Bioss, China) using the immunohistochemical staining kit (Santa, USA). Briefly, the sections were incubated with specific antibodies using a 1:200 dilution and subsequent detection with EnVison detection system (DAKO). Staining scores were quantified using IHS (IHS = A × B, A stands for positive cells percentage in five visions, B stands for staining intensity for positive cells) [24]. The H & E staining for tumor were referenced by histological and histochemical methods [25].

### Statistical analysis

The differences between groups were evaluated using Statistical Package for Social Science 20 (SPSS20.0). Statistical analysis was performed using a Student’s t test and survival differences among different groups of mice were evaluated with a log-rank test of the Kaplan-Meier survival curves. The statistical tests were two tailed and *p* values < 0.05 were considered to be statistically significant.

## Results

### Optimization of the mouse tumor model and experimental design

**Fig. 1:**
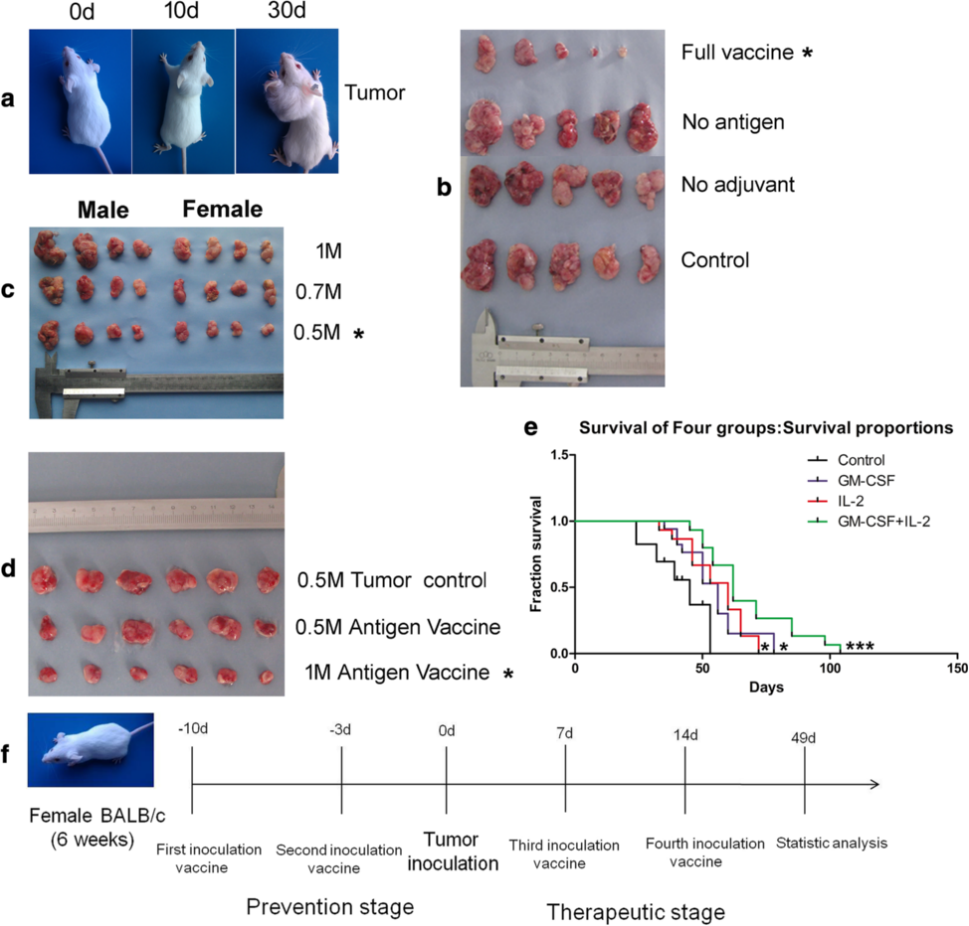
Standardization of the tumor model and its tentative mode of action The animals were vaccinated for two times before injected tumor cells during pre-experimental stage. Panel (**a**), shows the tumor growth after day 0, 10 and 30, as a result of subcutaneous injection of CT26.WT tumor cells into the neck of BALB/c mice. Panel (**b**), depicts the efficacy of cytokine adjuvant vaccine, where each group with 5 mice was vaccinated twice at interval of 7 and 3 days, before subcutaneous injection of CT26.WT cells. Panel (**c**), shows the comparison between male and female mice in terms of tumor uniformity. Panel (**d**), represents the standardization of inactivated antigen for effective anti-tumor response. M stand for million, * stand for Optimization. Panel (**e**), represents the effect of combination application compared to single group by observing survival period, which each group with 15 mice was vaccinated twice before subcutaneous injection of CT26.WT cells. *denotes *p* < 0.05, ***denotes *p* < 0.001. Panel (**f**), The experiment timeline showing the times of vaccinated. In order to establishing the tumor recurrence model stimulating the clinical status, the experiment was divided into prevention stage and therapeutic stage. The Prevention group was vaccinated vaccine for two times in prevention stage, the Treatment group was vaccinated vaccine for four times include prevention stage and therapeutic stage, and the Tumor group vaccinated inactivated antigen for control. All groups were inoculated tumor cells at the same time. Tumor inoculation was the initial point (day 0)

The schematic vaccine preparation and subsequent functional mechanisms for the induction of autologous T-cell anti-tumor response in a mouse colon cancer model is depicted in Additional file 1: Figure S1. Before analyzing treatment efficacy of the cytokine adjuvant vaccine, extensive standardization of different parameters to develop an ideal tumor model was applied, including the assessment of tumor formation time and volumes (Fig. 1a), specific anti-tumor effect of the vaccine (Fig. 1b), comparisons of tumor sizes between male and female mice (Fig. 1c), and optimized doses of inactivated tumor whole cell antigen in the vaccine (Fig. 1d). The results revealed that, there was no significant effect on the use of adjuvant or inactivated antigen alone, inactivated antigen obtained from 1 × 10^6^ tumor cells in the vaccine had the most significant effect on inhibition of tumor development in the female mice that subcutaneously injected with 5 × 10^5^ tumor cells. The tumor mass was generally established within 7–10 d post injection of the tumor cells. Based on these optimization steps, we compared the preliminary results of cytokine adjuvant vaccines between GM-CSF group, IL-2 group and GM-CSF + IL-2 group by observing the survival period. The results revealed that combined application extended the survival period and significantly inhibited the tumor growth compared to single group (Fig. 1e). Then we further examined treatment efficacy and mechanism of the cytokine adjuvant vaccination in a BALB/c mice colon cancer model. We divided the experimental procedures into the preventive and therapeutic stages, which may further strengthen anti-tumor effect during the course of tumor recurrence in clinical settings (Fig. 1f).

### Cytokine adjuvant vaccine enhanced anti-tumor immunity in the prevention and therapeutic models

**Fig. 2:**
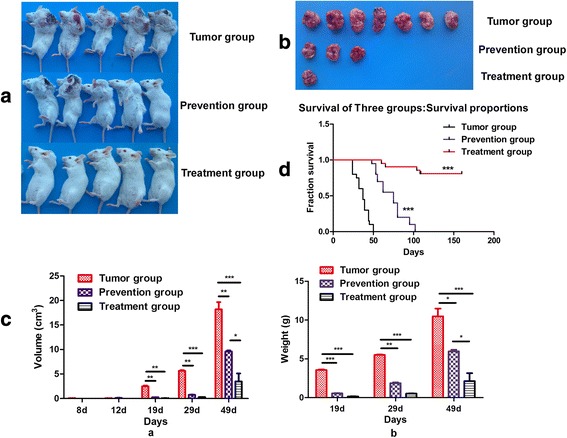
Preliminary effect of the cytokine adjuvant vaccine Panel (**a**), shows the pictures of mice with tumors at day 49 from three different groups. Panel (**b**), shows the pictures of tumors excised from each group of mice and subsequently these tumors were used to calculate the tumor inhibition rate. Panel (**c**), represents the comparison of tumor volume (left) and weight (right) between tumor, prevention and treatment group. Panel (**d**), depicts a survival curve analyzed with a log-rank test of Kaplan-Meier curves using mice from Tumor (control), Prevention and Treatment group, where 20 mice of each group were followed for a period of 160 d (Kaplan Meier, *p* < 0.001). ***denotes *p* < 0.001

Then, we set up the tumor model by subcutaneously injection of CT26.WT colon cancer cells (5 × 10^5^) at day 0. In order to determine if the vaccine had a preventive or therapeutic potential during tumor progression, firstly, we vaccinated the mice in the Prevention group twice, at 10 d (d − 10) and 3 d (d − 3) before tumor cell injection; secondly, in the Treatment group, respective twice vaccinations were given before and after tumor cell injection (d − 10, d − 3, d + 7, d + 14). The data showed that, whereas the weight of mice was not affected by vaccine inoculation (data not shown), the tumor growth as analyzed by tumor volumes and weights, was significantly inhibited in both groups of the vaccinated mice as compared to that in the controls. While 14 out of 14 (100 %) mice in the control group developed heavy tumors, only 6 out of 14 mice (42.8 %) and 2 out of 14 mice (14.3 %) in respective Prevention and Treatment groups had significantly less heavy tumors (Fig. 2a and b). As seen in Fig. 2c, Tumor group mice at 49 d post tumor cell injection had an average tumor volume and weight of 18.17 ± 3.94 cm^3^ and 10.48 ± 2.65 g respectively, Prevention group mice that were vaccinated two times had an average tumor volume and weight of 9.58 ± 0.36 cm^3^ and 5.97 ± 0.34 g whereas the Treatment group mice that were vaccinated four times had an average tumor volume 3.48 ± 0.43 cm^3^ and weight of 2.14 ± 0.21 g (*p* < 0.01). Furthermore, in comparison to the control group, the tumor inhibition rate was respective 57.1 % and 85.7 % in the Prevention and Treatment group. In addition, the survival rate was also assessed in the Control, Prevention and Treatment groups that each contained 20 mice, until 160 d post tumor cell injection. The data showed that mice in the Treatment group had greatly prolonged survival as compared with the Control group and Prevention group (Fig. 2d, *p* < 0.001). Taken together, these results indicated that vaccine was vaccinated four times including prevention and therapeutic stages were better than vaccinated two times. Then we discussed the mechanism between Tumor group and Treatment group for repeating experiment.

### Variation of CD4 + and CD8+ T cell populations in peripheral lymphoid organs

**Fig. 3:**
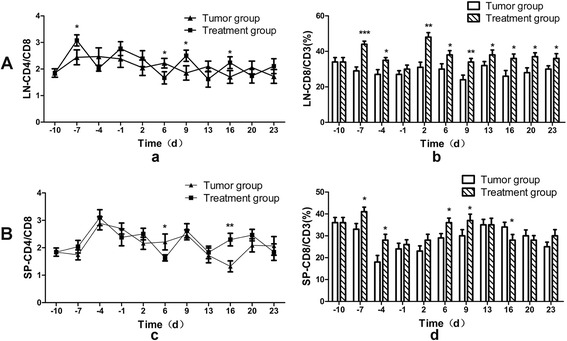
Variation of CD4+, CD8+ cell populations in peripheral lymphoid organs Panel **A**, depicts the CD4/CD8**+** and CD8/CD3**+** ratios as analyzed by flow cytometry analysis from the Lymph node, LN (**a**, **b**) Panel **B**, depicts the CD4/CD8**+** and CD8/CD3**+** ratios as analyzed by flow cytometry analysis from the Spleen, SP (**c**, **d**), isolated from Tumor (control) and Treatment group mice at an interval of 3–4 d. *denotes *p* < 0.05,**denotes *p* < 0.01, ***denotes *p* < 0.001

To further reveal variations of CD4+ and CD8+ T cells in the vaccinated and control mice, we stained the isolated CD3 + T cells from the draining LN and SP with specific anti-CD4 and -CD8 antibodies and examined their expression by FACS. The statistical results showed that the fluctuation of CD4/CD8 cell ratios during tumor progression after vaccination was significant and may reflect more rigorous and dynamic immune regulation and activity in the Treatment group (Fig. 3Aa and Bc). Moreover, ratios of CD8/CD3 cells in Treatment group was higher than the Tumor group in all time points in LN and in time points d-7 to d + 13 in SP (Fig. 3Ab and Bd). Then we found that the mice from the Treatment group were not able to form tumors when they were re-inoculated with the tumor cells (data not shown). The results indicated that T cell immune memory formed after vaccination protecting against tumor attacks. Furthermore, we detected the ability of the tumor-specific T cells.

### Vaccination enhanced the tumor-specific CTL responses

**Fig. 4:**
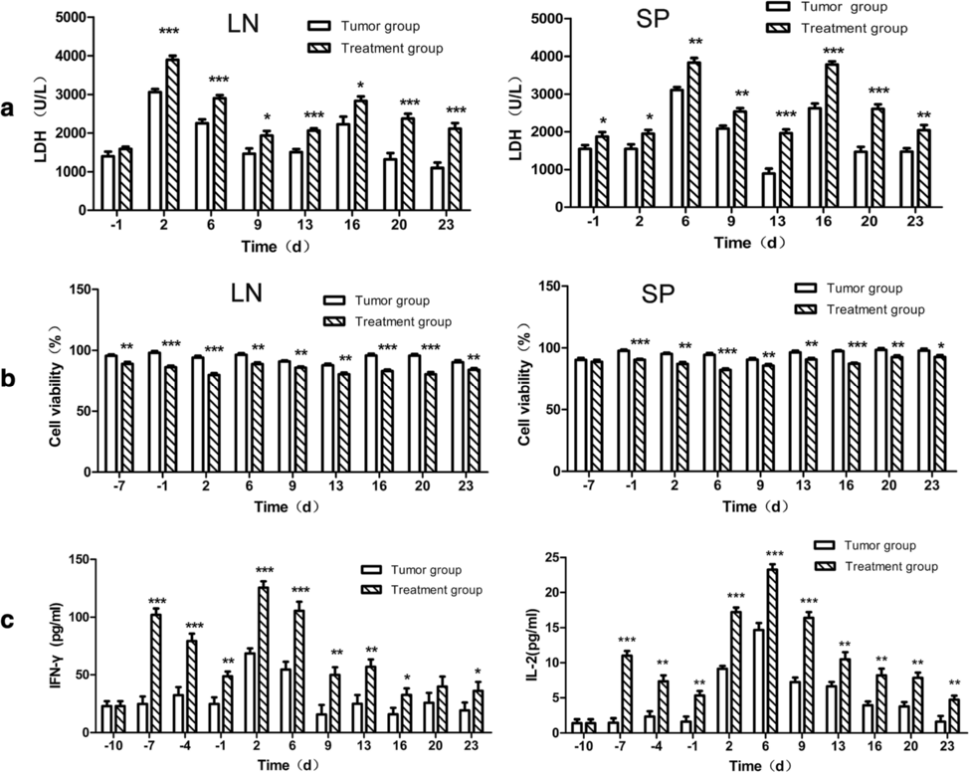
Tumor specific CTL activity in peripheral lymphoid organs and cytokines secretion in the serum The peripheral immune organs (LN and SP) were collected (3 mice/per group), and CD3+ T cells were isolated. These cells were incubated with CT26.WT tumor cells. Panel (**a**), represent the LDH release induced by T cells isolated from LN (left) and SP (right) of tumor control or treatment group mice. Panel (**b**), represents the CT26.WT tumor cell proliferation as measured by MTT assay post incubation with effector T cells at an *E: T* ration of 50:1. Panel (**c**), represents the secretion of cytokines measured by ELISA kit from the serum of tumor control and treatment group mice. Data are represented as mean ± SD. *denotes *p* < 0.05,**denotes *p* < 0.01, ***denotes *p* < 0.001

We further analyzed whether such a cytokine adjuvant vaccination enhanced tumor-specific CTL response. LDH assay was performed to analyze the cytotoxic effect. The results demonstrated that CD8 + T cells isolated from draining lymph nodes and the spleen of the vaccinated mice resulted in induction of higher LDH release by CT26.WT tumor cells in all time points in the Treatment group (Fig. 4a). In addition, the CTL activity was also examined by analyzing the target cell proliferation. The results showed that CT26.WT tumor cell proliferation was significantly reduced in samples that had CD8 + T cells from LN and SP of Treatment groups, at all time points as observed in Fig. 4b. These data suggested that CD8 + T cells isolated from the vaccinated mice were more effective in generating CTL response. Next, we analyzed the levels of cytokines IFN-γ, IL-2 and GM-CSF most probably produced by activated T cells in serum. The data from different time points suggested that, the Treatment group had significantly periodic increases in the IFN-γ and IL-2 levels as compared to the tumor group (Fig. 4c). Both cytokine levels peaked 2 d and 6 d post tumor cell inoculation and were significant higher in the vaccinated mice (*p* < 0.05). The levels of GM-CSF in serum in Treatment group were higher than the Tumor group which may stand for immune status for presenting antigen (Additional file 2: Figure S2). Collectively, this data suggested that the autolougous T-cell response were effectively elicited and significantly stronger in the Treatment group.

### Analysis on distribution of CD4, CD8, CD11c, CD80, CD86 and CD83 cell populations in lymph nodes and tumor tissues

**Fig. 5:**
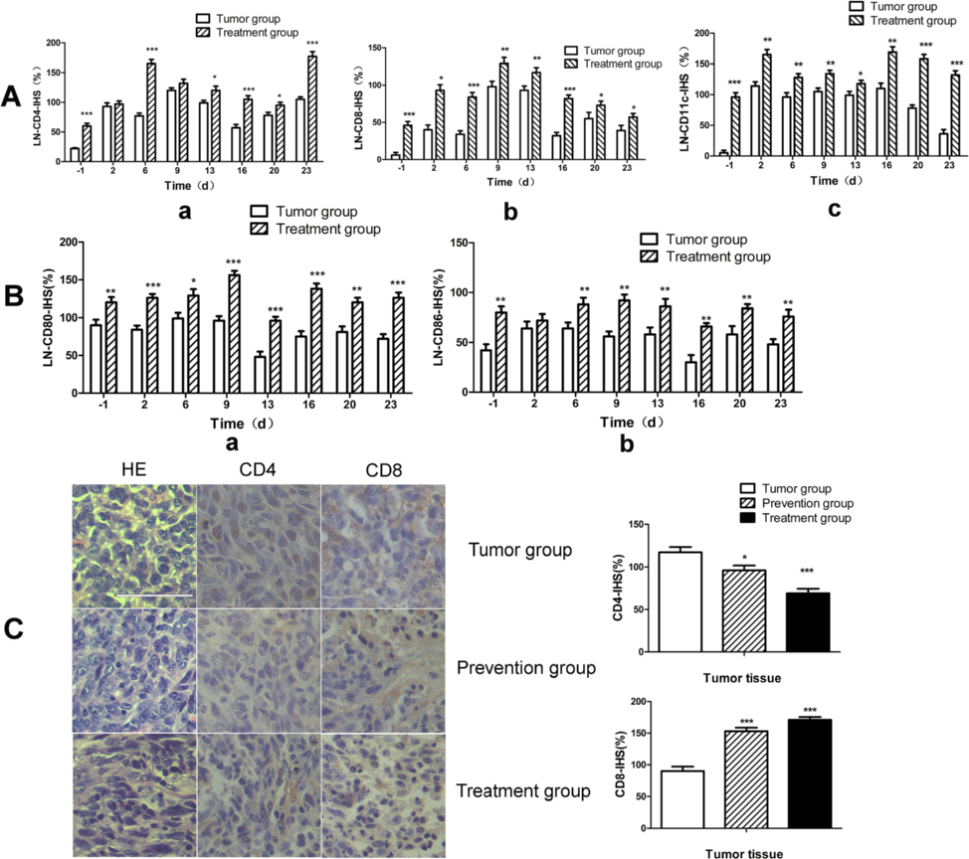
Distribution of CD4+, CD8+, CD11c+, CD80+ and CD86+ cell populations in Lymph node and tumor tissue Panel **A**, represents the expression of CD4 (**a**), CD8 (**b**) and CD11c (**c**) as analyzed by IHC staining of LN isolated at different time points from tumor control or treatment group mice. Panel **B**, represents the expression of CD80 (**a**) and CD86 (**b**) as analyzed by IHC staining of LN isolated at different time points from tumor control or treatment group mice. The expression of these molecules was calculated by IHS. Panel **C**, represents the HE and anti-CD4 and -CD8 IHC staining of tumor tissues collected from different groups. The results were observed in (40 × 10) horizon. Data are represented as mean ± SD. Bar = 50 μm. *denotes *p* < 0.05, **denotes *p* < 0.01, ***denotes *p* < 0.001

We next analyzed distribution of CD4, CD8, CD11c, CD80, CD86 and CD83 cells in the lymph nodes by IHC in order to confirm the FACS results (Fig. 5A, and B). Consistently, we confirmed that, as compared to the Tumor group, the samples in Treatment group had greatly higher expression of CD4, CD8 and CD11c, suggesting a strong immune response that induced by the vaccination in the Treatment group (Additional file 3: Figure S3). It is interesting to note that the expression of CD8 and CD11c in the Treatment group maintained significantly higher than that in the control group during the tested period of time (Fig. 5A b and c). The level of CD11c expression may represent the relative amount of mature DC and effective antigen presentation (Fig. 5Ac). In order to further confirm our results, specific surface molecules of CD80, CD86 and CD83 on DC cells were detected. The results showed that the different variable for three molecules (Fig. 5B and Additional file 4: Figure S4). There is no significant difference except for a few time points for the levels of CD83 during the Tumor group and Treatment group (Additional file 4: Figure S4C). However, the levels of CD80 were higher than the levels of CD86 in lymph nodes. Furthermore, the expressions of CD80 and CD86 in Treatment group were significantly higher than Tumor group especially on the back of a few time points the same as the results of CD11c (Fig. 5B a and b). The results suggested that GM-CSF could induce activation and maturation of DC and maintain at relevantly high levels during the course. These data suggested that the vaccine may induce the body to produce a stronger T cell response due to higher number of mature DCs in the lymph nodes.

Moreover, we compared distribution of CD4+ T& CD8+ T cells in the tumor tissues in different groups. Firstly, HE staining revealed that, in comparison to the tumor group whose tumors were more concrete, tumor tissue samples had significantly wider spread of tissue damage in the Treatment group, in which we further observed greater infiltration of the lymphocytes into the tumor tissues. Noticeably, the CD8+ T cells in the Prevention and Treatment groups tended to accumulated in the damaged area (Fig. 5C). However, the number of CD4+ cells in the Tumor group was higher than the Treatment group and were located around the tumor cells. These cells may aid tumor growth by behaving as regulatory T cells with an immuno-suppressive function. Such a phenomenon warrants further investigations.

## Discussion

In this study, we demonstrated safety and feasibility of a cytokine adjuvant vaccine in a mouse colorectal cancer model. To our best knowledge, this is the first study with a combination of the cytokines GM-CSF and IL-2 as a potent adjuvant of the vaccine that was applied in colon cancer models. Our data suggested that treatment with the cytokine adjuvant vaccine significantly delay or eradicate formation of colon cancer in this model. Thus, the vaccine and methodology may further used as cancer immunotherapeutic strategies to improve clinical recurrence after surgery.

It is shown that a sustained level of GM-CSF and IL-2 at the vaccination site is important for the activation of anti-tumor immune response [13], as GM-CSF acts to recruit DCs to present antigen, and IL-2 strengthens the T-cell response. Consistently, we showed that, if given sufficient amount in the colorectal cancer model, the vaccine may release GM-CSF and IL-2 in a good timing and effectively elicited an autologous T cells response, which achieved high efficacy in preventing from the tumor formation. Furthermore, there was a significant difference on the anti-tumor response in vivo between therapies in the prevention and treatment group, which adopted respective two and four times of the repeated vaccination. These indicate that the timing and times of vaccination procedures are important for an effective anti-tumor response. As approximately half of the colon cancer patients may suffer from tumor recurrence after surgery [26], our data imply that, combination of the inactivated resected tumor tissue as whole cell antigens and the cytokine adjuvant with optimized vaccination procedures on these patients may prevent from the tumor recurrence. Yet further investigations are highly warranted.

Besides significantly stronger CTL activity and prolonged survival rates in the treatment group, we observed greatly reduced ratios of weight/volume of the tumor mass in the treatment as compared to tumor group. The tumor mass in the treatment group mice were severely damaged and not as solid as that in the tumor group, in addition to our IHC analysis with an anti-CD8 antibody staining, we speculate that enhanced infiltration of the tumor specific T lymphocyte may contribute to such a phenomenon [27]. Furthermore, the levels of IFN-γ in serum play an important role in increased anti-tumor immune responses [28]. We found higher levels of IFN-γ in the serum of vaccinated mice. Moreover, IHC analysis of the lymph nodes showed higher levels of mature DC in the treatment group, which may provide specific antigen-MHC complexes for effective T cell response. Further IHC data with tumor tissues indicated that, the tumor group had a higher number of CD4+ T cells, whilst the number of CD8+ T cells was higher in the treatment group. Such results may show that the effector T cells may display either anti-tumor or tumor-suppressive effect in different groups of animals [29]. Nonetheless, more specified mechanisms of the vaccine in vivo and its application to other types of tumor models are ongoing in our lab; if the data are confirmed, we will further test the effect in relevant clinical studies.

## Conclusion

In summary, our data demonstrated that the cytokine (GM-CSF & IL-2) adjuvant vaccine had a significant anti-tumor effect in colon cancer of a mouse model. These results suggest that the effect maybe due to activating autologous T-cell response and an evaluation of such a vaccine in other cancer models and colon cancer patients is warranted to further test its immunotherapeutic efficacy.

## Additional files

Additional file 1: Figure S1. Provides schematic depiction of the vaccine formulation. (PDF 124 kb) Additional file 2: Figure S2. Provides the levels of GM-CSF in serum. (PDF 209 kb) Additional file 3: Figure S3. Provides the pictures for IHC staining for CD4, CD8 and CD11c in lymph nodes. (PDF 298 kb) Additional file 4: Figure S4. Provides the pictures for IHC staining for CD80, CD86 and CD83 in lymph nodes. (PDF 445 kb)

## Abbreviations

CTL: Cytotoxic T cells
FACS: Flow cytometry
GM-CSF: Granulocyte-macrophage colony-stimulating factor
HSA: Human serum albumin
IFN-γ: Interferon gamma
IHC: Immunohistochemical
IL-2: Interleukin-2
LDH: The lactic dehydrogenates
LN: Lymph nodes
MMC: Mitomycin C
SP: Spleen
