# Supplementary figures and images for: An effective cytokine adjuvant vaccine induces autologous T-cell response against colon cancer in an animal model
Source: BMC Immunol. 2016 Sep 26;17:31. doi: 10.1186/s12865-016-0172-x (PMC5037582; doi:10.1186/s12865-016-0172-x)

## Supplementary Figure S1

Fig.S1. Schematic depiction of the vaccine formulation

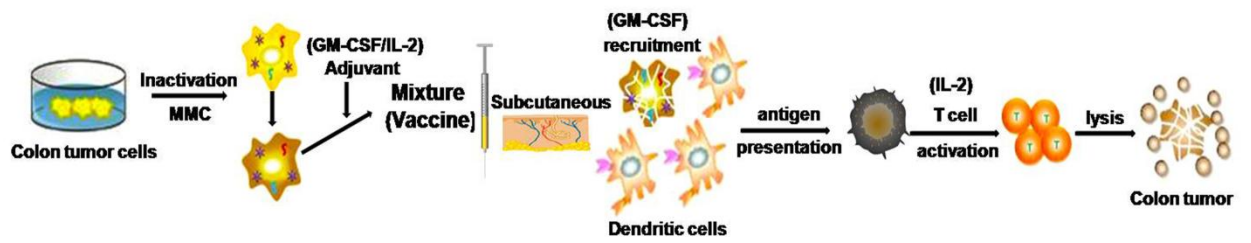

Supplement: Additional file 1: Figure S1. — Provides schematic depiction of the vaccine formulation. (PDF 124 kb) [file 12865_2016_172_MOESM1_ESM.pdf]
