# Supplementary material for: An effective cytokine adjuvant vaccine induces autologous T-cell response against colon cancer in an animal model
Source: BMC Immunol. 2016 Sep 26;17:31. doi: 10.1186/s12865-016-0172-x (PMC5037582; doi:10.1186/s12865-016-0172-x)

Supplementary Figure S2

Fig.S2. The levels of GM-CSF in serum It shows the levels of GM-CSF measured by ELISA kit from the serum of tumor control and treatment group mice. Data are represented as mean ± SD. *denotes $p < 0.05$， **denotes $p < 0.01$, ***denotes $p < 0.001$.

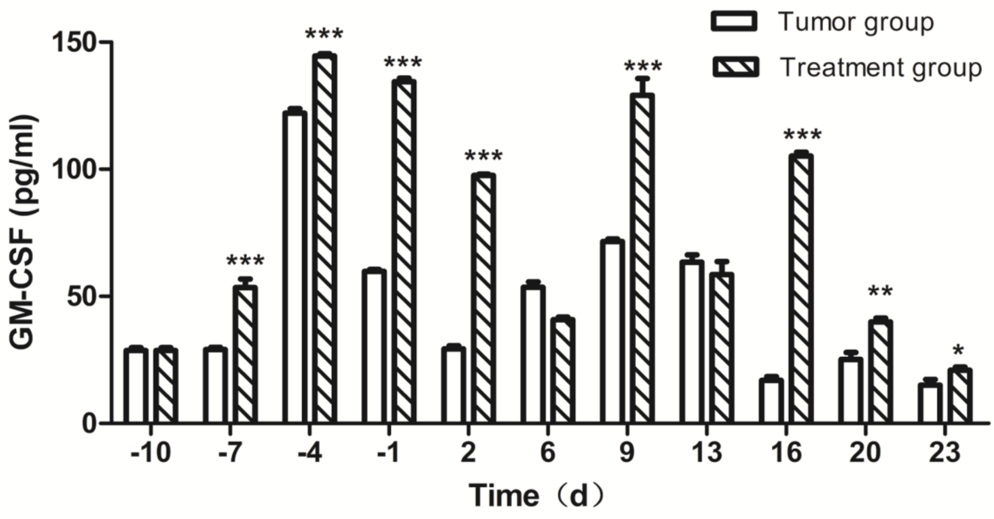

Supplement: Additional file 2: Figure S2. — Provides the levels of GM-CSF in serum. (PDF 209 kb) [file 12865_2016_172_MOESM2_ESM.pdf]
