# Supplementary material for: An effective cytokine adjuvant vaccine induces autologous T-cell response against colon cancer in an animal model
Source: BMC Immunol. 2016 Sep 26;17:31. doi: 10.1186/s12865-016-0172-x (PMC5037582; doi:10.1186/s12865-016-0172-x)

Supplementary Figure S3

Fig.S3. IHC staining for CD4, CD8 and CD11c in lymph nodes The sections of the LN collected at different time intervals from -1d to 23d period (1/per mouse and for 3 mice) from tumor control and treatment group mice, were stained with CD4 (panel A), CD8 (panel B) and CD11c (panel C) antibodies by IHC Kit. The results were observed in (40×10) horizon at time for -1d, 6d, 16d and 23d between tumor group and treatment group which stand for $p<0.001$.Bar=50μm.

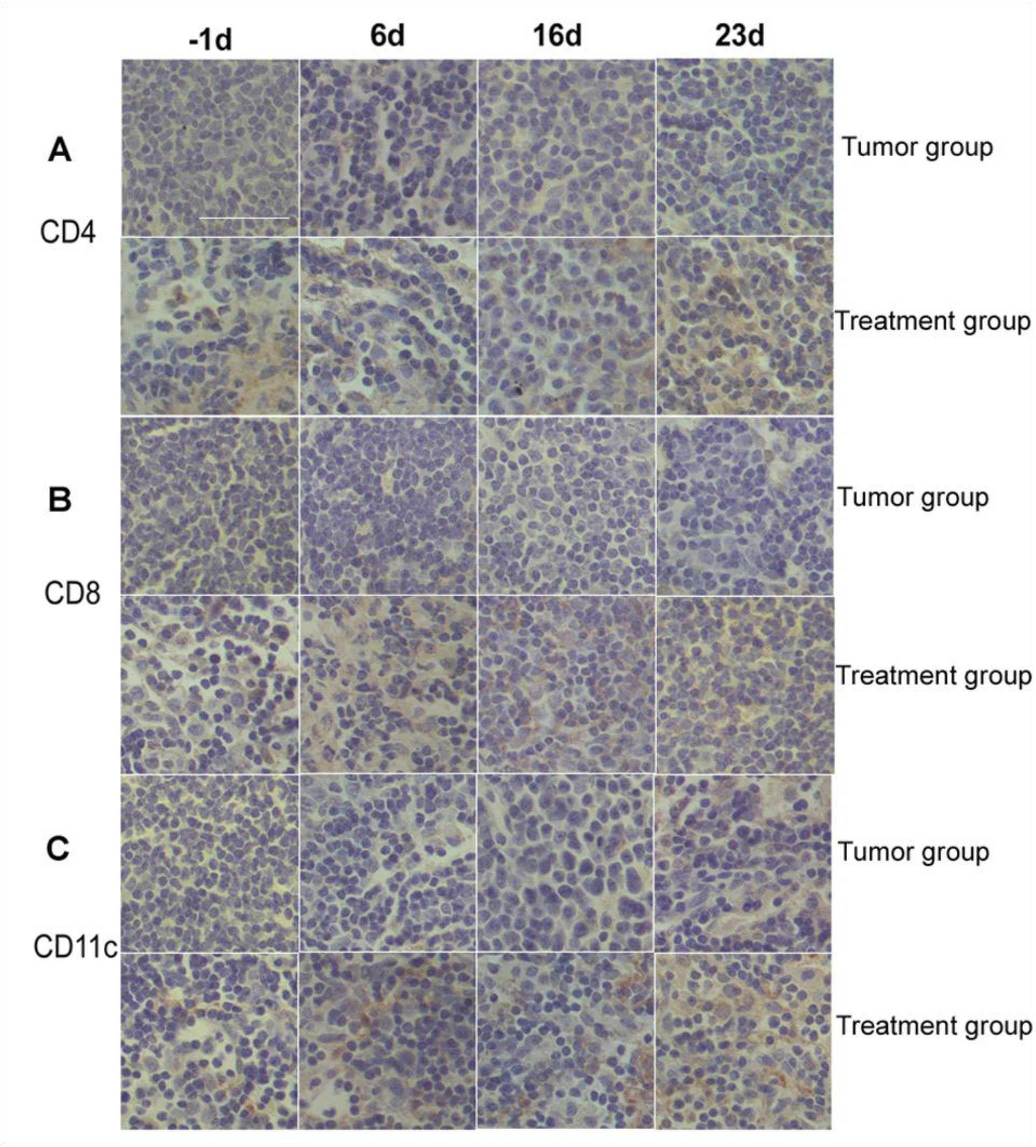

Supplement: Additional file 3: Figure S3. — Provides the pictures for IHC staining for CD4, CD8 and CD11c in lymph nodes. (PDF 298 kb) [file 12865_2016_172_MOESM3_ESM.pdf]
